# Chiroptical Properties and the Racemization of Pyrene and Tetrathiafulvalene-Substituted Allene: Substitution and Solvent Effects on Racemization in Tetrathiafulvalenylallene

**DOI:** 10.3390/molecules19032829

**Published:** 2014-03-04

**Authors:** Masashi Hasegawa, Seiya Iwata, Yasuto Sone, Junta Endo, Hideyo Matsuzawa, Yasuhiro Mazaki

**Affiliations:** Department of Chemistry, School of Science, Kitasato University, Sagamihara, Kanagawa 252-0373, Japan

**Keywords:** axis chirality, allene, tetrathiafulvalene, electronic circular dichroism, racemization

## Abstract

Dissymmetric 1,3-diphenylallene derivative **3** connected with 4,5-bis(methyl-thio)tetrathiafulvalenyl and 1-pyrenyl substituents was prepared and characterized. The molecular structure was determined by X-ray crystallographic analysis. Optical resolution was accomplished using a recycling chiral HPLC, and its chiroptical properties were examined with optical rotation and electronic circular dichroism (ECD) spectra. The title compound underwent photoracemization under daylight. This behavior was investigated in various solvents and compared with that of 1,3-bis(tetrathiafulvalenyl)allene (bis-TTF-allene) derivative **2**. The first-order rate plot of the intensity of the ECD spectra at a given time interval gave the rate of racemization. Mild racemization was observed in polar solvents, whereas a relatively fast rate was obtained in less polar solvents. In addition, the TTF groups of the allene also accelerate the racemization rate. These results suggest that the racemization mechanism occurs via a non-polar diradical structure.

## 1. Introduction

**Figure 1:**
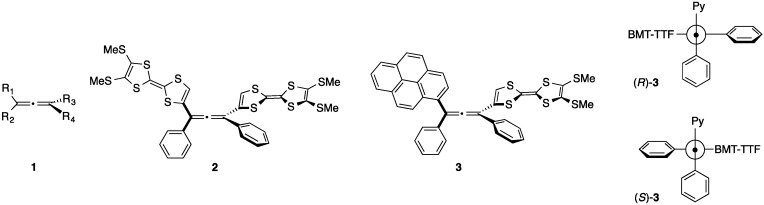
Allenes **1**–**3**.

Substituted allenes with axial chirality have been extensively studied as a class of chiral building blocks that are useful precursors for chirality transfer reactions, pharmaceutical agents, and materials possessing chiroptical properties [1,2,3,4,5,6]. Their definitive chirality imparts strong, reliable chiral effects in enantiocontrolled organic reactions and in polarized light-related optical properties. The axial chirality in the allene framework is defined by the substituents. When the combination of substituents is R^1^ ≠ R^2^ and R^3^ ≠ R^4^, allene **1** must be chiral (Figure 1). Typically, the chiral allene with substituents are stable toward racemization under ordinary circumstances because the rotation barrier of the allenic C=C=C bond is typically very large (*Δ*G^‡^ = 180 kJ/mol for 1,3-dimethylallenes) [7,8]. Therefore, while the racemization reaction occurs only under harsh conditions, such as irradiation by strong light, prolonged heating at very high temperatures, or addition of metal catalyst, chiral allenes are ordinarily obtained as chiral-persistent separable isomers after optical resolution or asymmetric synthesis [9,10,11,12,13,14,15,16].

Normally, π-conjugation decreases the racemization barrier; thus, some allenes with aryl or olefinic substituents are occasionally susceptible to racemization under mild conditions. Previously, Diederich and co-workers reported that certain chiral 1,3-diethynylallenes containing 4-*N*,*N*-dimethylanilyno (DMA) groups exhibited racemization in the presence of daylight [17]. This reactivity presumably stems from the electron donating ability of the terminal DMA groups. They also found that the photoisomerization in the shape-persistent macrocycles occurred on a chiral allene-ethynylene scaffold. In this case, the installed anthracene moieties may act as intramolecular sensitizers toward photoracemization [18]. More recently, we have developed 1,3-diphenyl-1,3-bis(tetrathiafulvalenyl)allene (**2**, Figure 1) and its derivatives as a new class of materials that have electrochromic chiroptical properties during their redox reaction. In the aforementioned system, the terminal tetrathiafulvalenes (TTFs) are effectively conjugated to the central allene, and this conjugation results in intensive chiroptical properties that show Cotton effect in the electronic circular dichroism (ECD) spectra depending on the redox state of each molecule [19]. However, allene **2** showed slow racemization in solution under daylight, whereas optical resolution by HPLC on a chiral stationary phase gave good separation of each enantiomer.

Many experiments and computational studies of the ground state and the excited state of a simple allene have been carefully executed in connection with the mechanistic investigation of the racemization reaction, the generation of a reactive carbene species, and the interactions within coherent laser light, among others [20,21]. Until now, the racemization mechanism of simple allenes with aliphatic substituents has been considered a process involving a planar triplet excited state of an ally diradical character after photosensitization of the solvent molecules [22]. Although a chiral allene installed into the rigid π-framework has great potential as a chiral source for versatile applications, a comprehensive understanding of the nature of racemization in aryl-substituted allenes has not been achieved.

To obtain further insight regarding chiral stability and the photoracemization reaction in chiral allenes installed into a large π-conjugation system, we have now designed a hetero-substituted allene, (*R*)/(*S*)-**3** (Figure 1) where the TTF and pyrene are connected at the 1 and 3 positions of the allene, respectively. Unlike compound **2**, replacing TTF into the pyrene unit may affect the stability of the axial chirality. Herein, we report the synthesis, structure, and chiroptical properties of **3**. Furthermore, the photoracemization reaction of **2** and **3** under daylight in various solvents has also been examined, and the rates of the racemization reaction were analyzed.

## 2. Results and Discussion

### 2.1. Synthesis of Pyrene and Tetrathiafulvalene-substituted Allene **3** and X-ray Determination

**Scheme 1:**
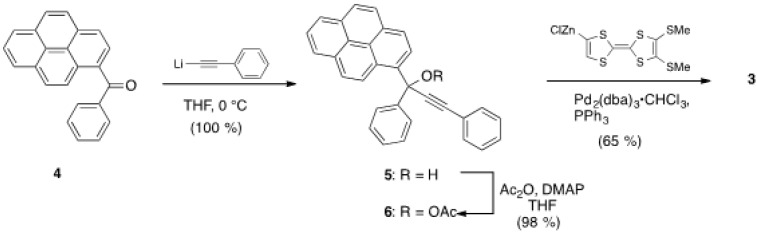
Synthesis of **3**.

An efficient route to connect the TTF moiety into the allenic framework was established by a palladium-catalyzed cross-coupling reaction as shown in Scheme 1 [23,24]. Starting with ketone **4**, prepared by Friedel-Crafts acylation of pyrene, the reaction with the lithiated acetylide of phenylacetylene in THF at low temperature afforded propargyl alcohol **5** quantitatively. The treatment of acetic anhydride with **5** in the presence of a catalytic amount of *N,N*-dimethyl-4-aminopyridine (DMAP) yielded acetyl ester **6** in 98% yield. Finally, addition of the organozinc intermediate derived from 4,5-bis(methylthio)tetrathiafulvalene (BMT-TTF) [25] into a mixture of **6** and palladium catalyst with PPh_3_ provided the allene compound **3** in moderate yield (65%). Compound **3** was fully characterized by ^1^H-, ^13^C-NMR, IR, MS, and elemental analysis. The chemical shift of **3** in ^13^C-NMR spectrum was found to be 208.2 ppm, suggesting a typical linear allenic framework. The vibrational stretching frequency of C=C=C was observed at 1919 cm^−1^ in the FT-IR spectrum, and it is also inconsistent with the bent structure.

**Figure 2:**
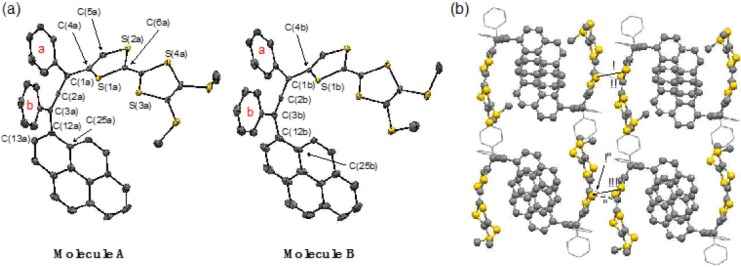
(**a**) ORTEP drawing of **3**. (**b**) Packing diagram of **3**. For clarity, the phenyl groups and the other atoms are displayed as the wireframe and the ball and stick model, respectively. Lines indicate selected intermolecular interactions. (i) S(1a)•••S(2a)^1^ 3.527 Å (1 = −x, 2−y, 1−z); (ii) C(6a)•••C(6a)^1^ 3.316 Å; (iii) S(1b)•••S(2b)^2^ 3.482 Å (2 = 1−x, 2−y, −z); iv) C(6b)•••C(6b)^2^ 3.247 Å; v) S(2b)•••C(6b)^2^ 3.466 Å.

Recrystallization of **3** from a CHCl_3_-hexane solution at 4 °C gave single crystals suitable for X-ray analysis. The molecular structure is shown in Figure 2. Compound **3** crystalizes with CHCl_3_ molecules in a triclinic system, space group P–1, and the unit cell contains two types of **3** (A and B in Figure 2a). Both molecular structures exhibit a typical allenic structure. The central C=C=C moiety is essentially linear (C(1a)–C(2a)–C(3a) = 173.5(5)° and C(1b)–C(2b)–C(3b) = 171.5(5)°). The dihedral angles of BMT-TTF against the central allene were found to be 21.4(6)° (for S(1a)–C(4a)–C(1a)–C(2a) in molecule A) and 21.6(6)° (for S(1b)–C(4b)–C(1b)–C(2b) in molecule B), whereas those of the pyrenyl groups were almost perpendicular to the central allene, whose dihedral angles were 89.26(6)° (for C(25a)–C(12a)–C(3a)–C(2a)) and 93.8(6)° (for C(25b)–C(12b)–C(3b)–C(2b)), respectively. Conversely, the dihedral angles of the two phenyl rings that are directly connected with the allenic skeleton were 46.0 (ring a) and 5.4° (ring b) for molecule A, and 49.8 (ring a) and 8.41° (ring b) for molecule B. Thus, the BMT-TTF moiety can reasonably conjugate to the central allene, while the pyrenyl group on the same side (ring a in Figure 2) is nearly perpendicular to the allene C=C bond. In the packing motif, each of the BMT-TTF parts dimerized in a face-to-face fashion with the equivalent part of the other enantiomer (Figure 2b). Several shorter distances within the sum of the van der Waals radii were found through S•••S and π-π interactions. Although the pyrenyl moiety also formed a dimeric motif in the crystal lattice, no intermolecular contacts were found between the parts.

### 2.2. Electrochemical Properties

**Figure 3:**
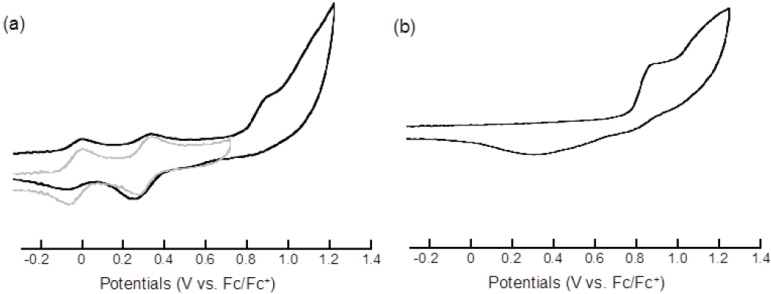
CV chart of (**a**) **3** (gray line: scan range −0.3 to 0.7 V; black line: scan range −0.3 to 1.2 V) and (**b**) pyrene.

The redox potentials obtained from the cyclic voltammetry (CV) analyses of **3** and its related compounds are summarized in Table 1. In the cyclic voltammogram of **3**, two reversible redox waves of *E*_1_^1/2^ = 0.00 and *E*_2_^1/2^ = 0.33 V (*vs.* Fc/Fc^+^) were observed within a range of −0.3–0.7 V (Figure 3a). Both redox waves are assigned to one-electron transfers for the successive formation of **3**^•+^ to **3^2+^**. Because these redox potentials are comparable to those of BMT-TTF **7**, the first and second oxidations correspond to the oxidation of the BMT-TTF fragment of **3**. The cyclic voltammogram of **3** exhibited two additional peaks when the scanning was performed with a wider potential range. Thus, the *E*_pa3_ of 0.92 V with anodic current and the *E*_pc3_ of 0.31 V with cathodic current were found as a pair of semi-reversible peaks. Compared with the CV of pyrene, these characteristic redox peaks can be assigned to the redox of the pyrene unit in **3**.

**Table 1:** Redox potentials of **3** and its related compounds *^a^*.

Compound	*E*_1_^1/2^	*E*_2_^1/2^	*E*_pa3_	*E*_pc3_
**3**	0.00	0.33	0.92 *^b^*	0.31 *^b^*^,*c*^
**7**	−0.02	0.34		
pyrene			0.92 *^b^*	0.33 *^b^*^,*c*^

*^a^* In CH_2_Cl_2_ containing 0.1 M*^ n^*Bu_4_NClO_4_ using a Pt working electrode and counter electrodes. The potentials were measured against an Ag/Ag^+^ electrode and converted to the value *vs*. Fc/Fc^+^. *^b^* Semi-reversible redox wave. *^c^* Shoulder peak.

### 2.3. Optical Resolution and ECD Spectra

The elution of racemic **3** with a hexane-CHCl_3_-EtOH solution (*v/v* = 40:10:0.2) on the chiral stationary phase (DAICEL Chiralpak IA-3) using a recycling chiral HPLC method efficiently provided optically active **3**. The resulting enantiomeric compounds (+)-**3** and (−)-**3** showed optical rotations of [α]_D_^24^ = +267 (*c* =0.0018) and −267 (*c* = 0.0015), respectively. To avoid racemization under light, the enantiomeric compounds were treated in the dark.

**Figure 4:**
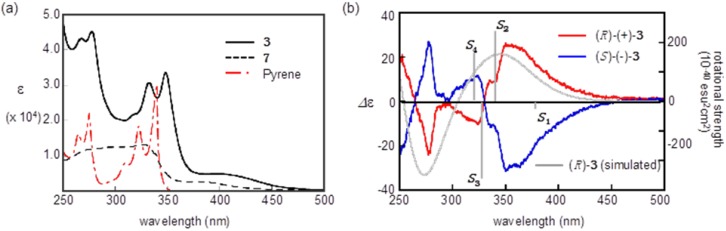
(**a**) UV-Vis spectra of **3** and its related compounds (**b**) ECD spectra of (*R*)-(+)-**3** and (*S*)-(−)-**3** together with the simulated ECD spectrum of (*R*)-**3** using a CAM-TD-DFT (CAM-B3LYP/6-311G(d,p)//B3LYP/6-31G(d,p)) calculation. The grey bars of *S*_1_–*S*_4_ indicate the selected rotational strength.

Figure 4 shows the electronic circular dichroism (ECD) spectra of the (+)-**3** and (−)-**3** isomers recorded in a hexane-CHCl_3_-EtOH solution (*v/v* = 40:10:0.2) as well as the absorption spectra of **3** and its related compounds in CHCl_3_. The UV-Vis absorption spectra of **3** in a CHCl_3_-hexane solution exhibit four absorption maxima at 268, 278, 332, and 348 nm with a shoulder absorption at 405 nm. From the spectra of **7** and pyrene, the four characteristic maxima observed in **3** can be assigned to the electronic transitions of the pyrenyl group on the allene, whereas the shoulder absorption can be attributed to the electronic transition of the BMT-TTF moiety. The ECD spectra of the (+)-**3** and (−)-**3** enantiomers exhibited mirror images with whose trend lines that appeared over the entire absorption range. An intensive Cotton effect was found at approximately 350 nm with long shoulder band tails up to approximately 450 nm, and the sign observed near 350 nm could be associated with the transition of the pyrenyl moieties. A positive sign from the first Cotton effect was observed in (+)-**3**, while a negative sign was observed from the first Cotton effect in (−)-**3**.

**Figure 5:**
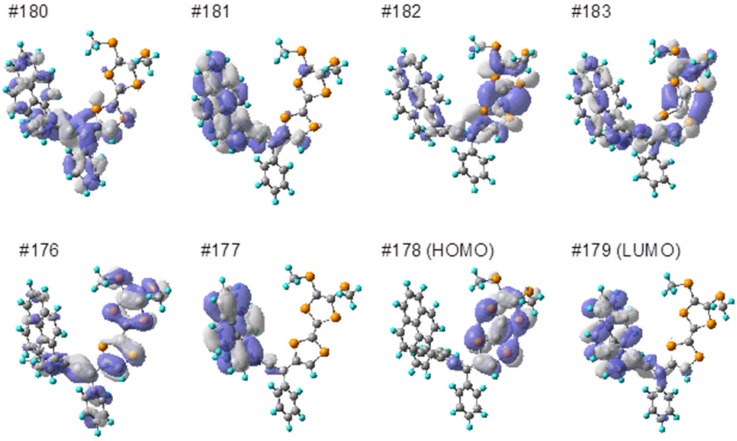
Molecular orbitals of **3**.

To the validation of the absolute configuration of (+)/(−)-**3**, a theoretical simulation of the electronic transition energies and rotational strengths was carried out by the employing time-depended Coulomb-attenuating density functional theory (TD-CAM-B3LYP/6-311G(d,p)) method following geometry optimization at the B3LYP/6-31G(d,p) level [26,27]. The optimized molecular geometry resembled the molecular structure observed in X-ray analysis: the pyrenyl group connects with the central allene almost perpendicularly, while the BMT-TTF moiety conjugates with the central allene. As shown in Figure 4b, the simulated curvature of the (*R*)-configuration of **3** is qualitatively satisfactory to represent the trend line of (+)-**3** for the sign of the first Cotton effect. The strong positive sign at 340 nm was calculated as the *S*_2_ transition that was associated with the mixing of electron transitions from the HOMO (#178) to the LUMO (#179) and from the HOMO-1 (#177) to the LUMO (#179), where the HOMO-1 and the LUMO mainly located at pyrenyl moiety (Figure 5). In addition, the electron transition found as *S*_1_ (at 379 nm), which was attributed to the transition of TTF moiety, also slightly found as the negative sign [28].

### 2.4. Racemization of **2** and **3** in Various Solvents

**Figure 6:**
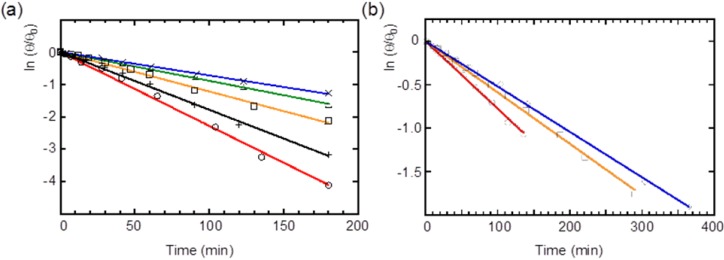
First-order rate plots for the intensity (at 355 nm) of the ECD spectra of (**a**) (*R*)-**2** and (**b**) (*R*)-**3** in benzene (circle and red line), MCH (plus and black line), CH_2_Cl_2_ (square and orange line), CH_2_Cl_2_–MeCN (4:1) (triangle and green line), and CH_2_Cl_2_–MeCN (1:1) (cross and blue line).

As mentioned previously, an allene framework with axial chirality is occasionally vulnerable to photoracemization at ambient temperature. The racemization behavior of **2** and **3** in various solvents was investigated in the daylight. A narrow range was scanned to obtain the ellipticity of the ECD spectra and the values were recorded in a quartz cell. Both **2** and **3** underwent photoracemization without decomposition, while the ECD spectra showed virtually no change in the dark. Figure 6 shows plots of the relative magnitude of ellipticity for **2** and **3** in various solvents at given time intervals. For **2**, relatively fast racemization proceeded in benzene and methylcyclohexane (MCH), while the more polar CH_2_Cl_2_ and a mixture of CH_2_Cl_2_-CH_3_CN exhibited slower racemization at the same concentration (0.5 M). Compound **3** also exhibited a similar behavior. Because these experiments were carried out in distillated, degassed solvents, a unimolecular reaction was anticipated. Therefore, the decrease in ellipticity can be analyzed as a first-order rate plot (Figure 6). Table 2 summarizes the kinetic data that were obtained for **2** and **3** from these analyses in various solvents. For both **2** and **3**, the polar solvents suppressed the racemization rates. On the contrary, the less polar solvents, benzene and MCH, greatly induced racemization rates. The rate of racemization for compound **3** in various solvents exhibited a similar tendency. However, it is noteworthy that the lower racemization rate was occurring in **3** rather than in **2**, which was found in an identical solvent.

**Table 2:** Racemization rate of **2** and **3** in various solvent.

Compound	Solvent	Rate (×10^−3^·min^−1^)	Half-life (min)
**2**	Benzene	22.8 ± 0.37	30
	MCH	17.8 ± 0.26	39
	CH_2_Cl_2_	12.1 ± 0.24	57
	CH_2_Cl_2_–MeCN (*v/v* = 4:1)	8.87 ± 0.08	78
	CH_2_Cl_2_–MeCN (*v/v* = 1:1)	7.18 ± 0.08	96
**3**	Benzene	7.78 ± 0.01	89
	CH_2_Cl_2_	5.91 ± 0.06	117
	CH_2_Cl_2_–MeCN (*v/v* = 1:1)	5.22 ± 0.04	133

A simple view of the photoisomerization of ethylenic double bonds (*i.e*., stilbene, 1,4-diphenyl-1,3-butadiene, *etc*.) is illustrated by the reduction of the bond order upon electronic excitation, followed by a twisting during the excited-state lifetime [29]. However, the detailed descriptions, even including multiplicity, are still complicated. Various influences, such as polarity, friction, viscosity, and heavy-atom effects of the solvent molecules, have been demonstrated in photoisomerization; however, it varies with substituents. Although the detailed mechanism of the racemization of **2** and **3** is still unclear, predominantly the solvent polarity rather than viscosity essentially affects to the rotation of the allene. Accelerating the racemization rates by decreasing polarity may imply a certain contribution of not dipolar but less polar character as exemplified by diradical species rather than dipolar species having charge-transfer character in excited state. The kinetic rate in MCH, that cannot be a photosensitizer, is comparable to that in benzene. It is also consistent with that supposition. Additionally, the enhancement of racemization in **2** compared with **3** suggests that the electron donating TTF moiety can act as a sensitizer to electronic excitation or as a stabilizer of the excited states, which both lead to the fastest racemization.

## 3. Experimental

### 3.1. General Information

^1^H and ^13^ C-NMR spectra were recorded on Bruker AVANCE-III-400 (Bruker Corporation, Billerica, MA, USA) (400 MHz for ^1^H, 100 MHz for ^13^C). Spectra are reported (in δ) referenced to internal Me_4_Si. Mass spectra were recorded on Thermo Scientific, Exactive Plus Orbitrap Mass Spectrometer (Thermo Fisher Scientific, Waltham, MA, USA) with atmospheric pressure chemical ionization (APCI) probe. IR spectra were recorded on JASCO FT/IR-610 spectrometer (JASCO, Hachioji, Japan). UV-Vis spectra were recorded on Hitachi U2800 spectrometer (Hitachi High-Technologies Corporation, Tokyo, Japan) using quartz cell. Melting points were determined with Yanaco melting point apparatus (Yanaco, Tokyo, Japan). Elemental analyses were performed on Perkin Elmer PE 2400-II CHNA/O analyzer (Perkin Elmer, Waltham, MA, USA). Optical resolution was carried out by recycling preparative HPLC (JAI Model LC-9204) (Daicel Corporation, Osaka, Japan) equipped with Daicel CHIRALPAK-IA3 column (20 × 250 mm). The optical rotations were measured with JASCO P-1300 spectropolarimeter (JASCO, Hachioji, Japan) in 1dm quartz cell at 24 °C. Electronic circular dichrimism (ECD) spectra were recorded on JASCO J-725 spectrodichrometer (JASCO, Hachioji, Japan). The spectra were combined after the baseline correction of each measurement. Cyclic voltammetry (CV) measurements were performed on Hokuto Denko HZ-5000 electrochemical analyzer (Hokuto Denko, Tokyo, Japan). X-ray crystallographic analysis was performed on Bruker SMART AXIS APEX-II (Bruker Corporation, Billerica, MA, USA). Column chromatography was carried out using Kanto chemical silica gel 60N, 60–210 m meshes. All solvents were dried by conventional procedures and distilled before use.

### 3.2. Synthesis of 1,3-Diphenyl-2-propyne-1-pyrenyl-1-ol **5**

To a solution of 1-phenylethyne (2.15 mL, 19.6 mmol) in THF (45 mL) was added dropwise *n*-C_4_H_9_Li (12.4 mL, 19.6 mmol) at 0 °C under an Ar atmosphere. After the mixture was stirred for 10 min, a solution of benzoylpyrene **4** (5.00 g, 16.3 mmol) in THF (150 mL) was added. The reaction mixture was stirred for 20 min at rt, and then poured into cracked ice. The product was extracted with CH_2_Cl_2_ and the resultant organic phase was washed with saturated brine and dried over MgSO_4_. Purification by column chromatography on silica gel with CH_2_Cl_2_ as eluent afforded **5** (6.65 g, quant.): light yellow powder; m.p.: 69.4–69.6 °C; MS (EI) *m/z* = 408 (M^+^); ^1^H-NMR (600 MHz, CDCl_3_) δ 8.68 (d, *J* = 7.8 Hz, 1H), 8.47 (d, *J* = 9.0 Hz, 1H), 8.17 (d, *J* = 9.0 Hz, 1H), 8.07–8.08 (m, 1H), 7.97–8.08 (m, 3H), 7.85–7.90 (m, 2H), 7.63–7.65 (m, 2H), 7.42–7.45 (m, 2H), 7.20–7.30 (m, 6H), 3.27 (s, 1H); ^13^C-NMR (150 MHz, CDCl_3_) δ 145.6, 137.1, 132.0, 131.9, 131.5, 130.6, 128.9, 128.8, 128.6, 128.4 (2C), 128.0, 127.7, 127.04, 127.03, 126.3, 126.08, 126.07, 125.58, 125.56, 125.0, 124.7, 124.5, 122.8, 92.4, 88.8, 75.7; IR (KBr) 3435, 3039, 2222, 1597, 1585, 1489, 1446 cm^−1^. Anal. Calcd for C_31_H_20_O: C, 91.15%; H, 4.94% Found: C, 90.84%; H, 4.71%.

### 3.3. Synthesis of 1,3-Diphenyl-2-propyne-1-pyrenylacetate (**6**)

To a solution of **5** (8.4 g, 21 mmol) and DMAP (250 mg, 2.0 mmol) in THF 40 mL was added dropwise Ac_2_O (9.6 mL) at 0 °C under an Ar atmosphere. The mixture was allowed to warm up to rt and stirred for over night. Then, the mixture was poured into iced water and the resulted white solid was collected by filtration. The white solid was used for next step without further purification due to instability of a solution of **6** (9.0 g, 98%): colorless solid; M.p. 169 °C (decomp.); MS (EI) *m/z* = 450 (M^+^); ^1^H-NMR (600 MHz, CDCl_3_) δ 8.75 (d, *J* = 8.1 Hz, 1H), 8.36 (d, *J* = 9.5 Hz, 1H), 8.24 (d, *J* = 8.1 Hz, 1H), 8.18 (dd, *J* = 7.5 and 1.1 Hz), 8.08–8.12 (m, 3H), 7.98 (t, *J* = 7.6 Hz, 1H), 7.90 (d, *J* = 9.5 Hz, 1H), 7.55–7.58 (m, 2H), 7.49–7.52 (m, 2H), 7.28–7.35 (m, 6H), 2.16 (s, 3H); ^13^C-NMR (150 MHz, CDCl_3_) δ 168.5, 143.5, 134.4, 132.3, 132.2, 131.6, 130.6, 129.0, 128.7, 128.6, 128.2, 127.8, 127.4, 127.3, 127.2, 126.4, 126.2, 125.8, 125.64, 125.58, 125.1, 145.5, 122.9, 91.5, 89.1, 22.1; IR (KBr) 3053, 2226, 1741, 1597, 1587, 1489, 1365, 1230 cm^−1^; Anal. Calcd for C_33_H_22_O_2_: C, 87.98%; H, 4.92% Found: C, 87.54%; H, 4.85%.

### 3.4. Synthesis of the Allene **3**

To a solution of 4,5-bis(methylthio)tetrathiafulvalene (394 mg, 1.3 mmol) in THF (8 mL) was added dropwise *n*-C_4_H_9_Li (0.81 mL, 1.33 mmol) at −78 °C under an Ar atmosphere. After stirring for 1.5 h, a suspension of anhydrous ZnCl_2_ (182 mg, 1.3 mmol), which was dried at 200 °C *in vacuo* for 3 h, in THF 4 mL) was added dropwise into the mixture at −65 °C. The mixture was stirred for 2 h at the same temperature, followed by warming up to −10 °C. Then, a solution of **6** (200 mg, 0.44 mmol), Pd_2_(dba)_3_•CHCl_3_ (79 mg, 0.27 mmol), and PPh_3_ (79 mg, 0.27 mmol) in THF (4 mL) was added dropwise at −10 °C, and the resulting mixture was further stirred for 14 h at rt. The mixture was poured into saturated aqueous NH_4_Cl solution, and the products were extracted by CH_2_Cl_2_. The resultant crude product was purified by column chromatography on silica gel with CH_2_Cl_2_/hexane (*v/v* = 1) as the eluent. Recrystallization from CH_2_Cl_2_/hexane gave **1** as yellow crystals (200 mg, 65%): M.p. 160–161 °C. MS (EI) *m/z* = 686 (M^+^); ^1^H-NMR (600 MHz, CDCl_3_) δ 8.25 (d, *J* = 7.8 Hz, 1H), 8.21 (dd, *J* = 7.8 and 1.2 Hz), 8.15 (d, *J* = 7.8 Hz), 8.12–8.16 (m, 3H), 8.09 (d, *J* = 8.8 Hz, 1H), 8.0 (t, *J* = 7.5 Hz, 1H), 7.97 (d, *J* = 9.0 Hz, 1H), 7.43–7.45 (m, 2H), 7.34–7.39 (m, 3H), 7.24–7.33 (m, 5H), 6.13 (s, 1H), 2.44 (s, 3H), 2.41 (s, 3H); ^13^C-NMR (150 MHz, CDCl_3_) δ 208.2, 136.1, 134.5, 133.5, 131.33, 131.31, 131.0, 130.3, 129.5, 128.8 (2C), 128.77, 128.75 (2C), 128.4, 127.90, 127.87, 127.81, 127.77, 127.7, 127.6, 127.4, 126.1, 125.7, 125.34, 125.26, 125.09, 125.06, 124.7, 116.6, 111.7, 107.4, 106.8, 19.27, 19.24; UV-Vis (5.0 × 10^−3^ M, CHCl_3_): λ_max_ (ε) 268 (43200), 278 (45200), 332 (30700), 348 (33600), 405sh nm; IR (KBr) 3041, 2918, 1919, 1491, 1444 cm^−1^; Anal. Calcd for C_39_H_26_S_6_: C, 68.18%; H, 3.81% Found: C, 68.12%; H, 4.17%.

### 3.5. X-ray Crystallographic Study of **3**

X-ray crystallographic measurement of **3** was made by using graphite-monochromated MoKα (λ = 0.71069 Å) radiation. The unit cell parameters and the diffraction intensities were collected with Bruker SMART AXIS APEX-II at −100 °C. The crystal structure was solved by using SHELXS-97 and refined by using the full matrix least-squares method included in SHELXL-97. All non-hydrogen atoms were refined anisotropically, and hydrogen atoms were refined isotropically. The crystal lattice contains (*R*)-**3** and (*S*)-**3** together with two CHCl_3_ molecules in P-1 space group. Crystal data for **3**: 2(C_39_H_18_S_6_)•2(CHCl_3_), *M*r = 1612.65, Triclinic, P-1 (#2), *α* = 9.7195(8)Å, *b* = 15.5891(12) Å, *c* = 24.0137(19) Å, *α*= 92.685(1)°, *β*= 15.5891(12)°, *γ*= 24.0196(1)°, *V* = 3599.7(5) Å^3^, *Z* = 2, *d*_calc_ = 1.488 g/cm^3^, *T* = 173 K, *R*_1_ = 0.0737 (*I* > 2σ(*I*)), *wR*_2_ = 0.1848 (*I* > 2σ(*I*)), gof = 1.012. CCDC 982197 contains the supplementary crystallographic data for this compound. The data can be obtained free of charge from The Cambridge Crystallographic Data Centre via www.ccdc.cam.ac.uk/data_request/cif.

### 3.6. Computational Details

All quantum chemical calculations were performed with the Gaussian 09 (Revision C.01) program. The molecular structures of **3** was estimated by theoretical calculations at the RB3LYP/6-31G(d,p) level, and its minimum energies were confirmed by frequency calculations. The standard orientation of the optimized structures of **3** was summarized in Table S2. Excitation energy was computed using Coulomb-attenuating density functional theory (CAM-TD–B3LYP) with 6-31G(d,p) basis set in RB3LYP/6-31G(d,p) optimized geometry (nstate = 64).

## 4. Conclusions

Herein, we have described the synthesis and characterization of 1-[4,5-bis(methylthio)-tetrathiafulvalenyl]-3-pyrenyl-1,3-diphenylallene (**3**) as a dissymmetric framework. X-ray analysis indicated the effective conjugation of TTF with the central allene, whereas the pyrenyl group was almost perpendicular. CV measurements revealed that **3** underwent two one-electron reversible redox processes responsible for the oxidation of TTF and the successive one-electron semi-reversible redox reaction facilitated by the pyrene unit. Optical resolution of the enantiomers was achieved using chiral HPLC. The chiroptical properties were also investigated by ECD spectra, and the obtained spectra enabled validation of their absolute configurations by TD-DFT calculations. Both optically pure allenes of (+)/(−)-**3** showed intensive Cotton effects in their ECD spectra over their entire absorption range. The absolute configurations of the enantiomers were assigned the by validation of the electronic transition energies calculated with TD-DFT method at the CAM-B3LYP/6-311G(d,p) level. The photoracemization behaviors of (*R*)-**2** and (*R*)-**3** were examined under daylight using an analysis of the relative magnitude of ellipticity in the ECD spectra at given time intervals. An increase in the reaction rate with decreasing solvent polarity was observed, presumably because of the non-polar excited state or intermediates. Furthermore, the number of TTF groups also affects quick racemization. The detailed mechanism, especially the photoracemization dynamics by monochromated light irradiation is currently being elucidated. We hope to use this information to design new, valuable π-extended optically pure allenes without racemization under ambient conditions. 
