# Supplementary material for: Chiroptical Properties and the Racemization of Pyrene and Tetrathiafulvalene-Substituted Allene: Substitution and Solvent Effects on Racemization in Tetrathiafulvalenylallene
Source: Molecules. 2014 Mar 4;19(3):2829–41. doi: 10.3390/molecules19032829 (PMC6271782; doi:10.3390/molecules19032829)

Supporting Information

Contents

Figure S1. (**a**) ^{1}H-NMR and (**b**) ^{13}C NMR spectra of **3** p. S2

Figure S2. ^{13}C-NMR Spectra of **3** (105~140 ppm) p. S3

Table S1. Detailed X-ray parameters of **3** p. S4

Figure S3. Chiral HPLC chart of **3** p. S5

Figure S4. Optimized structure of **3** p. S5

Table S2. Molecular coordinate of optimized structure of **3**-(A) pp. S6–S7

TD-DFT calculations of 3 and MO diagram p. S8

Figure S1. (**a**) ^{1}H-NMR spectra and (**b**) ^{1}H-^{1}H-Cosy spectra of **3**.

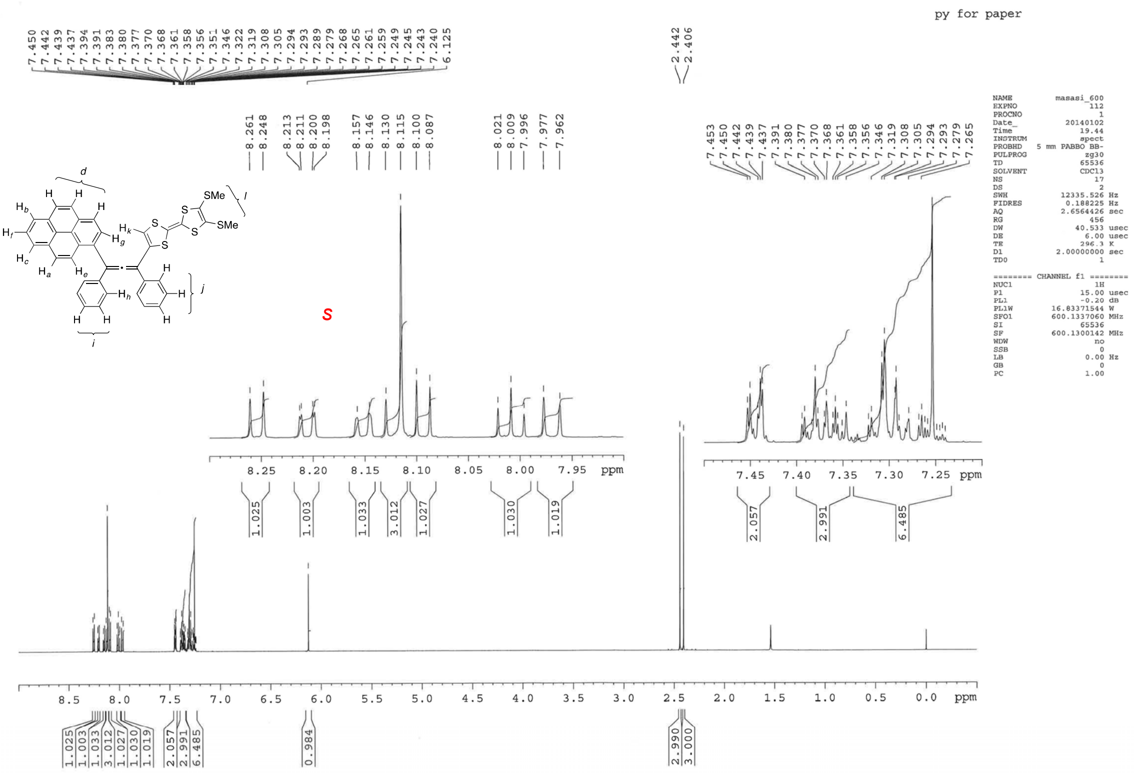

(**a**)

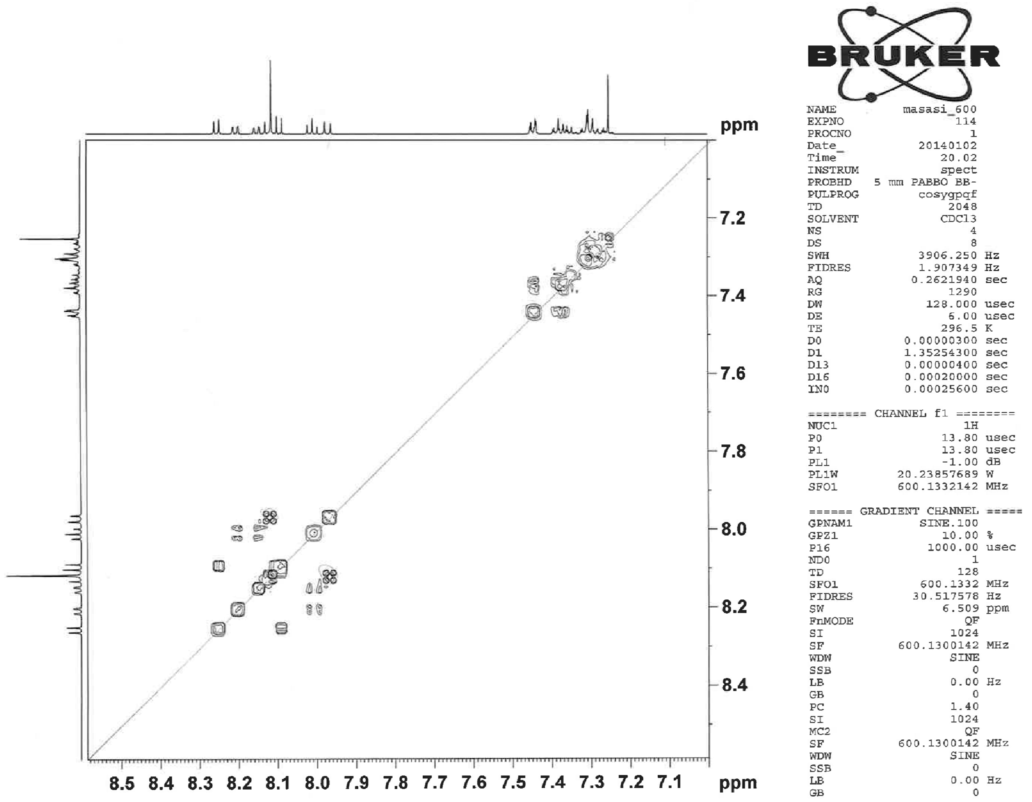

(**b**)

Figure S2. ^{13}C-NMR Spectra of **3**. (**a**) 105~140 ppm; (**b**) 120~220 ppm.

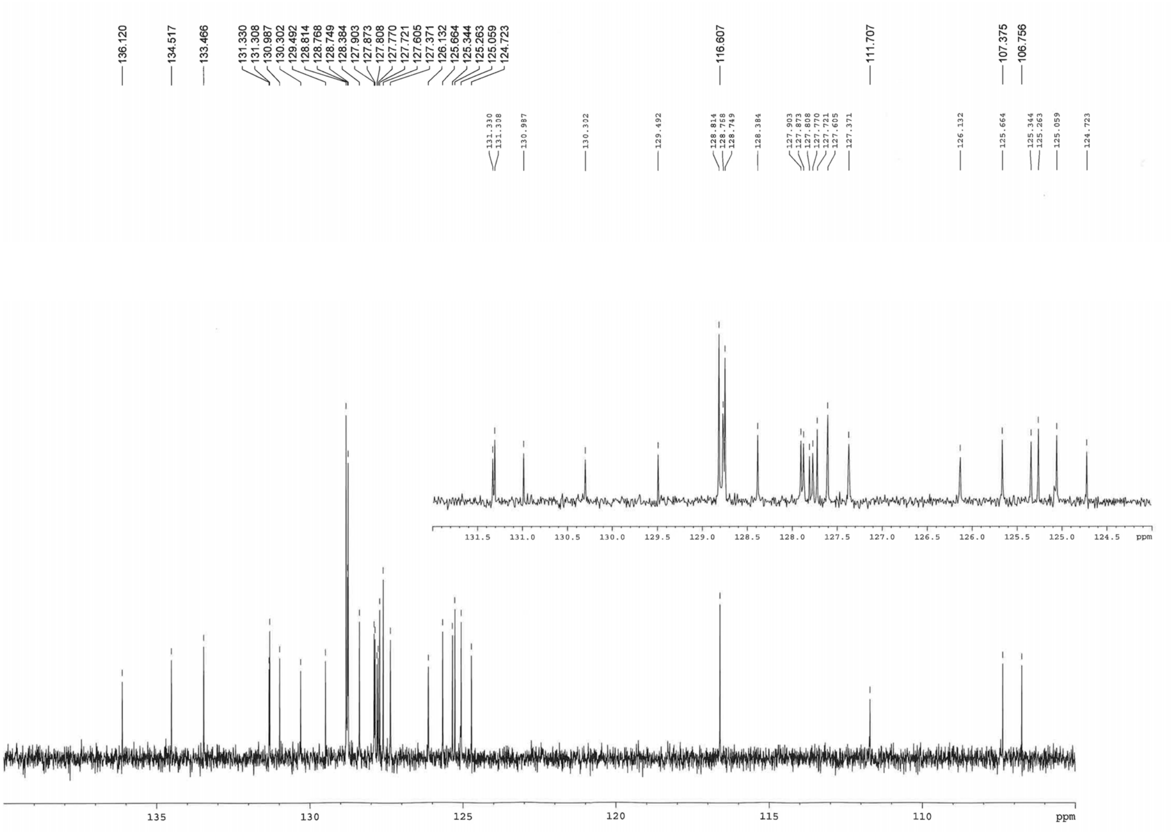

(**a**)

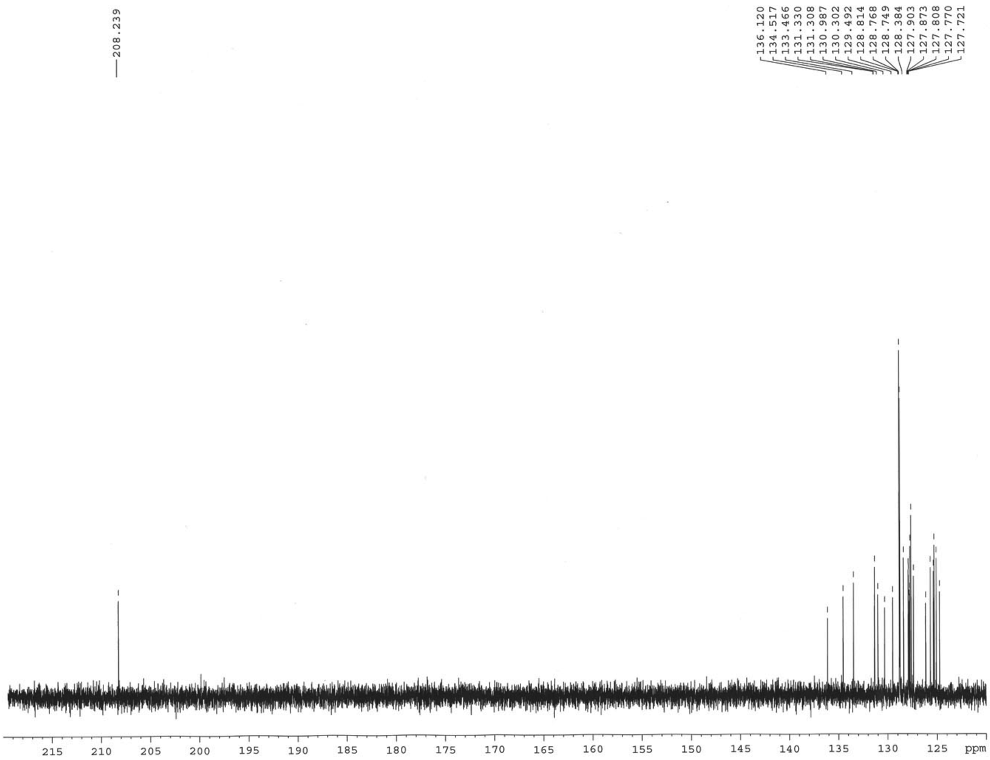

(**b**)

Table S1. Detailed X-ray parameters of **3**.

**Identification code**	**3**
Empirical formula	$C_{80}H_{54}S_{12}Cl_6$
Moiety formula	$2(C_{39}H_{18}S_6)$•$2(CHCl_3)$
Formula weight	1612.65
Temperature	173
Wavelength	0.71073 Å
Crystal system	Triclinic
Space group	P-1 (#14)
Unit cell dimensions	α = 9.7195(8)Å
	b = 15.5891(12) Å
	c = 24.0137(19) Å
	α = 92.685(1) °
	β= 15.5891(12) °
	γ= 24.0196(1) °
Volume	3599.7(5) $Å^3$
Z	2
Density (calcd.)	1.488 g/cm^3
Absorption coefficient	0.634 mm^{-1}
F(000)	1656.0
Crystal size	0.20 × 0.10 × 0.10 mm^3
Theta range for data	1.53 to 27.50 °
Index ranges	$-11 \leq h \leq 12$
	$-19 \leq k \leq 20$
	$-30 \leq 1 \leq 25$
Reflections collected	20,121
Independent reflections	15,369 [R(int) = 0.0284]
Refinement method	Full-matrix least-squares on
Data/restraints/parameters	15,369/0/889
Goodness-of-fit on F^2	1.012
Final R indices [$I > 2$ (I)]	R_1 = 0.0737, wR_2 = 0.1848
[all data]	R_1 = 0.1325, wR_2 = 0.2301
CCDC deposition No.	982197

Figure S3. Chiral HPLC chart of **3**.

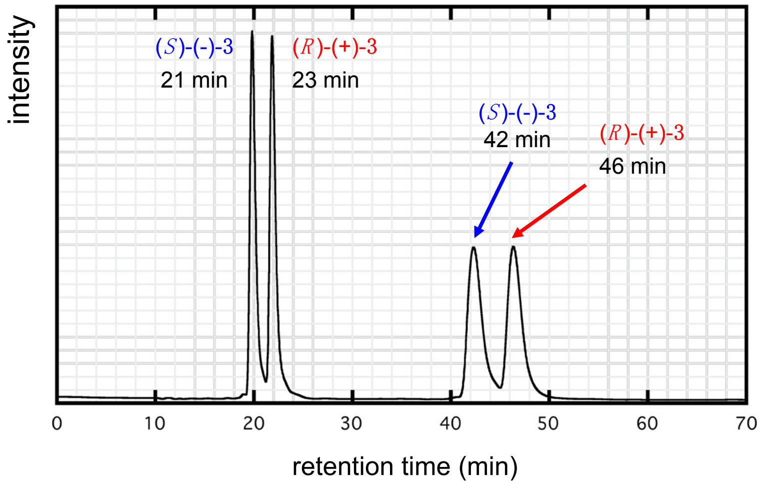

Column: CHIRALPAK® IA-3 (ϕ 20 × 250 mm); Eluent: Hexane/$CHCl_3$/EtOH = 40:10:0.2 (*v/v*); Flow Rate: 6.0 mL/min; Temperature: 25 °C; Detection: 270 nm.

DFT Calculation of 3

The geometry optimization was performed by DFT calculation with B3LYP/6-31G(d,p) basis set. The optimized structures of **3**-A and **3**-B in Figure S4 are obtained from X-ray analysis (Molecule A and Molecule B, respectively). Other conformers of **3**-C, **3**-D, and **3**-E are obtained from the initial structures described by Z-matrix format. Among these calculations, only the conformers that the pyrene is perpendicular to the central allene were found. Their optimized structures were confirmed by further frequency calculations. Both optimized structures basically adopted a similar conformation except for the orientation of the SMe groups (Figure S4). The geometry having the lowest energy (Molecule A) was treated further TD-CAM-B3LYP calculation.

Figure S4. Optimized structure of **3**.

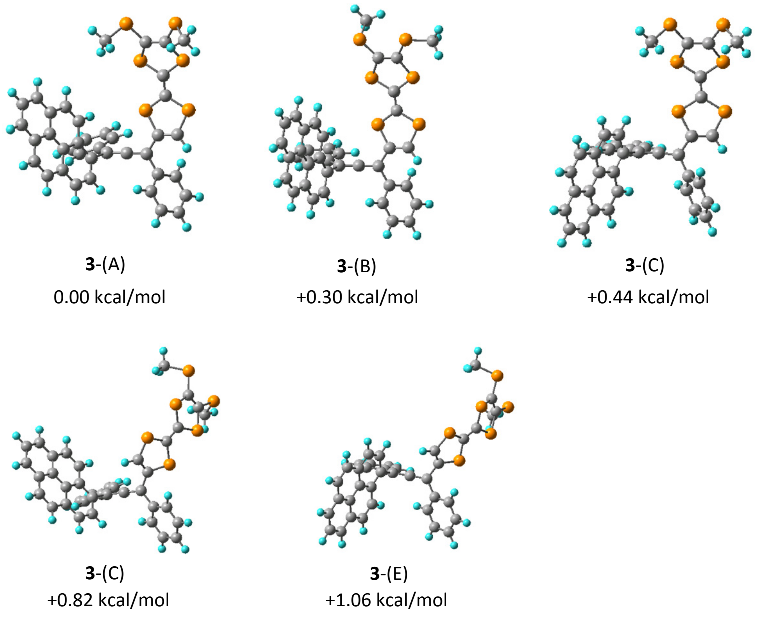

Table S2. Molecular coordinate of optimized structure of **3**-(A).

Center Number	Atomic Number	Atomic Type	Coordinates (Angstroms) X	Y	Z
1	16	0	−1.003464	−1.512706	0.691662
2	16	0	−3.564304	0.494352	1.366856
3	16	0	−5.370118	−1.278889	−0.149001
4	16	0	−2.826630	−3.290895	−0.860358
5	16	0	−5.361827	2.933026	1.106832
6	16	0	−7.452907	0.878761	−0.647482
7	6	0	−3.698099	−0.984290	0.385558
8	6	0	−2.660800	−1.801730	0.106912
9	6	0	1.870075	−4.041623	−0.988373
10	6	0	−1.098664	−3.544635	−1.031373
11	1	0	−0.783419	−4.350128	−1.681649
12	6	0	1.221025	−2.851437	−0.343190
13	6	0	−0.249068	−2.759162	−0.340991
14	6	0	−5.032966	1.251095	0.679072
15	6	0	−5.862799	0.436292	−0.018228
16	6	0	1.564995	−5.345230	−0.567044
17	1	0	0.842735	−5.494992	0.229496
18	6	0	2.813530	−3.863163	−2.009752
19	1	0	3.042286	−2.857096	−2.346978
20	6	0	2.194194	−6.442884	−1.153396
21	1	0	1.954649	−7.445530	−0.811294
22	6	0	3.133784	−6.255367	−2.169163
23	1	0	3.621963	−7.111501	−2.625260
24	6	0	3.441339	−4.962667	−2.595358
25	1	0	4.166999	−4.808489	−3.388696
26	6	0	−3.923240	3.795236	0.356488
27	1	0	−2.981473	3.415966	0.754822
28	1	0	−4.034945	4.847059	0.629185
29	1	0	−3.938973	3.698108	−0.730040
30	6	0	−7.258656	0.516001	−2.438047
31	1	0	−6.516031	1.177362	−2.886943
32	1	0	−8.236058	0.709058	−2.885856
33	1	0	−6.985077	−0.526726	−2.603723
34	6	0	2.741769	−0.995611	0.761885
35	6	0	3.108759	−1.031378	2.209529
36	6	0	1.971494	−1.912743	0.210497
37	6	0	1.838944	1.841819	0.914596
38	1	0	1.383077	1.092127	1.550978
39	6	0	2.367510	−1.797247	3.126062
40	1	0	1.499163	−2.348580	2.778451
41	6	0	2.729785	−1.846023	4.468638
42	1	0	2.140931	−2.439805	5.161789
43	6	0	4.217494	−0.310998	2.680589

Table S2. *Cont.*

Center Number	Atomic Number	Atomic Type	Coordinates (Angstroms)		
			X	Y	Z
44	1	0	4.801543	0.285694	1.988184
45	6	0	2.858569	1.431465	−0.013282
46	6	0	3.838008	−1.128119	4.926446
47	1	0	4.117418	−1.163644	5.975254
48	6	0	3.297426	0.089549	−0.121429
49	6	0	4.578242	−0.361390	4.026994
50	1	0	5.441251	0.201187	4.371243
51	6	0	3.436938	2.423199	−0.866880
52	6	0	3.166400	6.109007	−1.507313
53	1	0	3.609575	6.863750	−2.151371
54	6	0	3.595239	4.777168	−1.621586
55	6	0	2.009549	4.160149	0.168123
56	6	0	4.602554	4.375722	−2.564695
57	1	0	5.040184	5.135305	−3.206991
58	6	0	2.186416	6.466425	−0.582952
59	1	0	1.868077	7.502084	−0.508580
60	6	0	1.438465	3.140124	1.000973
61	1	0	0.666882	3.425372	1.711230
62	6	0	1.612404	5.505070	0.245785
63	1	0	0.848667	5.789634	0.964554
64	6	0	4.438960	2.059376	−1.818382
65	6	0	3.014105	3.784342	−0.773829
66	6	0	5.003782	3.078976	−2.658800
67	1	0	5.765660	2.789201	−3.377557
68	6	0	4.275305	−0.243235	−1.066632
69	1	0	4.603977	−1.275746	−1.132891
70	6	0	4.838557	0.717836	−1.899784
71	1	0	5.600974	0.432435	−2.619528

Calculated by B3LYP/6−31G(d,p).

TD-DFT calculations of 3 and MO diagram

Table S3. Selected electronic transition for **3** ((TD-CAM-B3LYP/6-311G(d,p)//B3LYP/6-31G(d,p)).

Excited State	Energy	Oscillator Strengths	Rotational Strength in cgs (10^{-40}esu^{2}cm^2)	Nature	
S_1	379 nm<br>3.270 eV	0.0059	−0.883	178 ->183	0.62864
S_2	340 nm<br>3.645 eV	0.3634	254	178 ->179<br>177 ->179	0.41451<br>0.32502
S_3	326 nm<br>3.799 eV	0.2012	−266	177 ->179<br>178 ->182	0.48724<br>0.37257
S_4	320 nm<br>3.877 eV	0.1328	154	178 ->179<br>177 ->179	0.31356<br>0.30927

Figure S5. MO diagram of **3**.

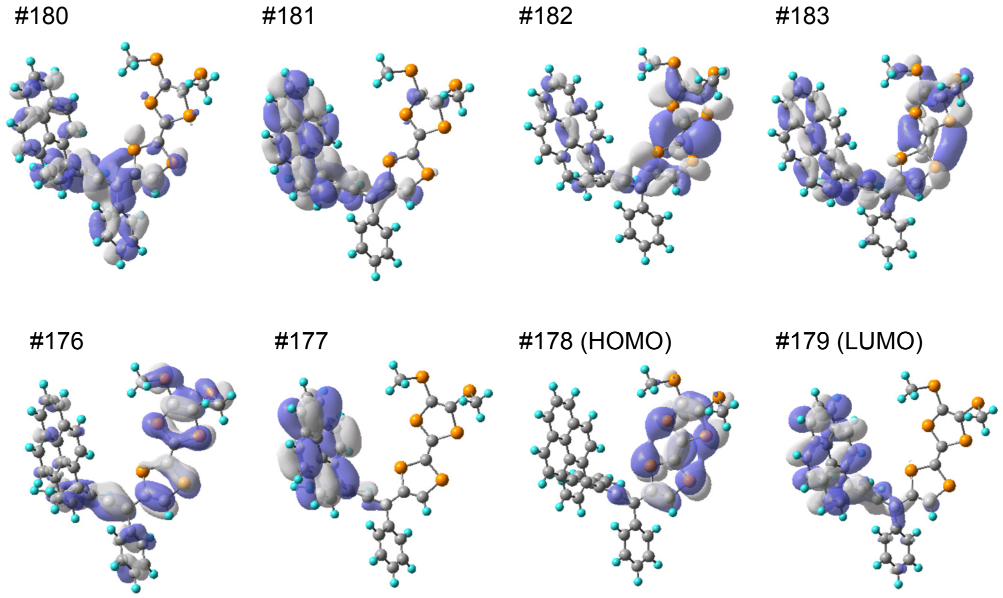

Supplement: Supplementary file 1 [file molecules-19-02829-s001.pdf]
